# Correction: Inotuzumab ozogamicin in pediatric patients with relapsed/refractory acute lymphoblastic leukemia

**DOI:** 10.1038/s41375-019-0426-8

**Published:** 2019-03-07

**Authors:** Deepa Bhojwani, Richard Sposto, Nirali N. Shah, Vilmarie Rodriguez, Constance Yuan, Maryalice Stetler-Stevenson, Maureen M. O’Brien, Jennifer L. McNeer, Amrana Quereshi, Aurelie Cabannes, Paul Schlegel, Claudia Rossig, Luciano Dalla-Pozza, Keith August, Sarah Alexander, Jean-Pierre Bourquin, Michel Zwaan, Elizabeth A. Raetz, Mignon L. Loh, Susan R. Rheingold

**Affiliations:** 1 Children’s Hospital Los Angeles and Keck School of Medicine, University of Southern California, Los Angeles, CA USA; 2 National Cancer Institute, Bethesda, MD USA; 3 Mayo Clinic, Rochester, MN USA; 4 Cincinnati Children’s Hospital Medical Center, University of Cincinnati School of Medicine, Cincinnati, OH USA; 5 University of Chicago Medicine Comer Children’s Hospital, Chicago, IL USA; 6 Oxford University Hospitals, Oxford, UK; 7 University Hospital Saint-Louis, Paris, France; 8 University Children’s Hospital of Würzburg, Würzburg, Germany; 9 University Children’s Hospital of Münster, Münster, Germany; 10 Children’s Hospital at West-mead, Sydney, Australia; 11 Children’s Mercy Hospital, Kansas City, MO, USA; 12 The Hospital for Sick Children, Toronto, Canada; 13 University Children’s Hospital, Zurich, Switzerland; 14 Erasmus MC, University Medical Center, Rotterdam, the Netherlands; 15 New York University Langone Medical Center, New York, NY USA; 16 Benioff Children’s Hospital and the Helen Diller Family Comprehensive Cancer Center, University of California San Francisco, San Francisco, CA USA; 17 Childrens Hospital of Philadelphia, University of Pennsylvania, Philadelphia, PA USA

**Keywords:** Cancer immunotherapy, Acute lymphocytic leukaemia

**Correction to:** **Leukemia** (2018);

10.1038/s41375-018-0265-z;

published online 28 September 2018

In the original version of this Article the following authors were omitted:

• Claudia Rossig (University Children’s Hospital of Münster, Münster, Germany)

• Paul Schlegel (University Children’s Hospital of Würzburg, Würzburg, Germany)

• Jean-Pierre Bourquin (University Children’s Hospital, Switzerland)

• Aurelie Cabannes (University Hospital Saint-Louis, Paris, France)

• Luciano Dalla-Pozza (Children’s Hospital at Westmead, Sydney, Australia)

• Sarah Alexander (The Hospital for Sick Children, Toronto, Canada)

• Michel Zwaan (Erasmus MC, University Medical Center, Rotterdam, the Netherlands)

• Amrana Quereshi (Oxford University Hospitals, UK)

• Keith August (Children’s Mercy Hospital, Kansas City, MO, USA)

All authors agree to the inclusion of these added authors

The acknowledgments section has also been updated to read:

We thank the research coordinators and following physicians at pediatric cancer centers for contributing data to this project: Prashant Hiwarkar and Jayashree Motwani, Birmingham Women’s and Children’s Hospital, UK; Kelly Maloney, Children’s Hospital of Colorado, USA; Mylene Bassal, Children’s Hospital of Eastern Ontario, Canada; Yoav Messinger and Joanna Perkins, Children’s Hospital of Minnesota, USA; Van Huynh, Children’s Hospital of Orange County, USA; Richard Ho, Children’s Hospital at Vanderbilt, USA; Joanne Chuah and Jessa Morales, Children’s Hospital at Westmead, Australia; Donald Wells, Dell Children’s Hospital, USA; Nicolas Boissel, Hospital Saint-Louis, France; Tannie Huang, Kaiser Permanente, USA; Stacey Marjerrison, McMaster Children’s Hospital, Canada; William Carroll and Joanna Pierro, New York University Langone Medical Center, USA; Ajay Vora, Sheffield Children’s Hospital, UK; Donna Lancaster, The Royal Marsden Hospital, UK; Lucie Šrámková, University Hospital Motol, Czech Republic; Chatchawin Assanasen, University of Texas Health Science Center, San Antonio, USA; Rupert Handgretinger, University of Tübingen, Germany.

This has now been corrected in both the PDF and HTML versions of the Article.

